# Supplementary material for: Patterns of Intron Gain and Loss in Fungi
Source: PLoS Biol. 2004 Nov 30;2(12):e422. doi: 10.1371/journal.pbio.0020422 (PMC532390; doi:10.1371/journal.pbio.0020422)
Supplement: Table S1 — Also available at http://genes.mit.edu/NielsenEtAl/. (4.3 MB ZIP). [file pbio.0020422.st001.zip › NielsenEtAl/html/927.html]

AN0913.1.NCU09192.1.MG08368.1.FG05154.1


```
 CLUSTAL W (1.82) Multiple Sequence Alignments - Introns Inserted


Sequence 1: AN0913.1	306 aa
Sequence 2: FG05154.1	310 aa
Sequence 3: NCU09192.1	259 aa
Sequence 4: MG08368.1	311 aa
Alignment Length: 319 aa
Number Identitical Residues: 143 aa
Alignment Score (without introns) 6823


MG08368.1 	MSGPRTRKQAAREAAAGEHAPTSNGDSGANAQELL-------VKKDEFVENENIFAFWPN
NCU09192.1	---------------------MPATEKVSRG--------------KDAEPRENVYLFVPN
FG05154.1 	MS-PRTRRQAGKASNASSSESEPEVRSNGNGVAHR------AADVSDDAPRENIFLFYPN
AN0913.1  	MS-ARTRRQ--KAALAAQTEGSDDVSSTSNGTIQRPPKQSRSASPEDDGVTENVYLFAPN
          	 : . :  .    : :..        . ...    ....: : . .:    **:: * **

MG08368.1 	IIG1YIRVVLALISLYYMPLHPRTCTLLYSISCLLDALDGYLARLFEQSTRFGAVLDMVT
NCU09192.1	LIG1YSRIILAVASLYYMPLHPRTCSLLYSISCLLDALDGYAARYLDQSTRFGAVLDMVT
FG05154.1 	IIG1YARIVLAIASLYYMPLHPRTCSFLYSVSCLLDALDGYAARIYEQSTRFGAVLDMVT
AN0913.1  	IIG1YVRVVLAIASLYYMPLHPRTCSLLYSVSCLLDALDGYAARYYNQSTTFGAVLDMVT
          	:** * *::**: ************::***:********** **  :*** *********

MG08368.1 	DRCTTACMLVFLSSAFPRWAIVFQGLISLDFASHYIHMYTTLAMGGSGQSHKNVDRSRSW
NCU09192.1	DRCTTACLLVFLASAFPRWSIVFQGLIALDMASHYMHMYATLAMGGSDESHKNVDKSRSF
FG05154.1 	DRCTTACLIVFLSSAFPRWAIVFQGLIALDLASHYMHMYATLVVSGSNASHKSIDESKNW
AN0913.1  	DRCTTACLLVFLSSAWPRWALVFQSLISLDMASHYMHMYATLSMGGANQSHKKIDSSRSW
          	*******::***:**:***::***.**:**:****:***:** :.*:. ***.:* *:.:

MG08368.1 	ILNQYYTNK0VVLFVFCFLNEAFFIALYLLSFSSPLLSPTLLGQVPPGAAEEILKGNREV
NCU09192.1	ILNLYYTNR0TVLFLCCALNEVFFIALYLLSFSSPLLSPGLLE--QVPQIKQ--------
FG05154.1 	LLRLYYTNK0GVLFALCALNELFFIALYLLCFSSPLVSPYLIK--PIEIVGAELQAGAQV
AN0913.1  	VLYLYYNSR0TVLFICCALNELFFIGLYLLSFSSPTLSSSLLQ--PPQGVTVGGTPPPPA
          	:*  **..:  ***  * *** ***.****.**** :*. *:                  

MG08368.1 	DSSLLGQIFKNPYSAGALELARANKM0DSTIPWIVAIVSFPVMLGKQFINVVQFIKASKW
NCU09192.1	---------ANPYSAAALELARANKM0DSTVPWIIAIVSFPVMMLKQIINVVQLVKASRW
FG05154.1 	NTSILAQIFPDPFSPAALELARANKM~DSTVPWIIAGISFPFMFMKQLINCVQLYNASQG
AN0913.1  	STS----VFASPWSAGALELARANKI~DSFWPWVITAVSFPVMAFKQFVNVVQLVKASNW
          	.::       .*:*..*********: **  **::: :***.*  **::* **: :**. 

MG08368.1 	LAEGDLEARRAQGLPRPKKTI-
NCU09192.1	LAEGDLEARRERKKTR------
FG05154.1 	LAEIDLKTRREQGLPRPKPKKA
AN0913.1  	LVEGDLDNRRKARKARDN----
          	*.* **. **    .* .
```
